# Oncoviral Infections and Small Extracellular Vesicles

**DOI:** 10.3390/v16081291

**Published:** 2024-08-13

**Authors:** Łukasz Ważny, Theresa L. Whiteside, Monika Pietrowska

**Affiliations:** 1 Maria Sklodowska-Curie National Research Institute of Oncology, 44-102 Gliwice, Poland; s88267@365.sum.edu.pl; 2 UPMC Hillman Cancer Center, University of Pittsburgh Cancer Institute, Pittsburgh, PA 15232, USA; whitesidetl@upmc.edu; 3 Department of Pathology, University of Pittsburgh School of Medicine, Pittsburgh, PA 15213, USA; 4 Department of Immunology, University of Pittsburgh School of Medicine, Pittsburgh, PA 15213, USA

**Keywords:** oncoviruses, small extracellular vesicles (sEV), exosomes, viral infections

## Abstract

Small extracellular vesicles (sEV) are small membrane-bound nanovesicles with a size range below 200 nm that are released by all types of cells. sEV carry a diverse cargo of proteins, lipids, glycans, and nucleic acids that mimic the content of producer cells. sEV mediate intercellular communication and play a key role in a broad variety of physiological and pathological conditions. Recently, numerous reports have emerged examining the role of sEV in viral infections. A significant number of similarities in the sEV biogenesis pathways and the replication cycles of viruses suggest that sEV might influence the course of viral infections in diverse ways. Besides directly modulating virus propagation by transporting the viral cargo (complete virions, proteins, RNA, and DNA), sEV can also modify the host antiviral response and increase the susceptibility of cells to infection. The network of mutual interactions is particularly complex in the case of oncogenic viruses, deserving special consideration because of its significance in cancer progression. This review summarizes the current knowledge of interactions between sEV and oncogenic viruses, focusing on sEV abilities to modulate the carcinogenic properties of oncoviruses.

## 1. Introduction

The rapid development of biological sciences that took place in the 20th century, including the development of electron microscopy (EM), has enabled the emergence of research on extracellular vesicles (EVs). Traditionally, the 1946 publication by Chargaff and West, which describes the procoagulant activity in a pellet from blood plasma obtained by ultracentrifugation, is considered as the first reported association of the thromboplastic factor with EVs [1,2]. It was not until 1967 that Peter Wolf described the detection by EM of small microparticles in plasma, referred to as “platelet dust” [3]. Research conducted in subsequent years has not only allowed for the identification of different types of EVs (among them, small extracellular vesicles; sEV aka exosomes) but has also led to the understanding that EVs are not a product of cell breakdown like apoptotic bodies. EVs are produced by live cells and represent a key element of intercellular communication, performing a variety of physiological and pathological functions [4]. The field of EV research has been characterized by dynamic developments in recent years, with an increasing number of publications focusing on the impact of EVs on viral infections.

The current definition of EVs identifies them as lipid bilayer membrane-enclosed nanovesicles released by all types of cells. Unlike cells, EVs cannot replicate independently and lack a nucleus [5]. EVs exhibit significant diversity in size, cellular origin, and biogenesis. Because of this heterogeneity, the nomenclature of EVs is not entirely clear, although the most commonly used terminology divides them into small EVs (sEV; with a diameter <200 nm) and large EVs (lEVs; with a diameter >200 nm) [5]. Further, based on their biogenesis, EVs can be divided into exosomes (EXOs) derived from the endocytic cell compartment; ectosomes (microvesicles, MVs) produced by pinching off from the surface of cell membrane; and apoptotic bodies [5,6]. The International Society for Extracellular Vesicles (ISEV) has recently issued recommendations that all vesicles smaller than 200 nm that originate from the endocytic cell compartment of producer cells should be referred to as “sEV”. [5]. EVs are present in all human biological fluids [7], including blood [8,9], urine [10], saliva [11,12], tears [13], ejaculate [14], bronchial fluid [15], cerebrospinal fluid [16], peritoneal fluid [17], amniotic fluid [18], milk [19], and bile [20]. In this review, we use the terms “sEV” or “exosomes” based on the vesicle size as well as cellular origin.

The biogenesis of sEV begins with the endocytosis of external materials and the formation of early endosomes in the cytosol of a producer cell. The endosomal membrane undergoes re-invagination, forming intraluminal vesicles (ILVs) within the multivesicular bodies (MVBs). Subsequently, the fusion of MVBs with the cellular membrane leads to the release of exosomes into the intercellular space [21]. The biogenesis of exosomes is tightly controlled by the endosomal sorting complex required for transport (ESCRT), which regulates the selection of exosomal cargo as well as ILV formation. Alternative ESCRT-independent pathways of exosome biosynthesis have also been described [22,23].

As mediators of intercellular communication, sEV are involved in a wide range of physiological functions. The role of sEV in stimulating both innate and acquired immune responses has been previously demonstrated [24,25]. sEV can fulfill these functions by transferring antigens, cytokines, and enzymes, as well as transcription factors and cytotoxic proteins, all of which can induce transcriptional and/or translational changes in recipient cells. An interesting example of such reprogramming is offered by sEV released by antigen-presenting cells (APCs) that can enhance antibacterial responses against *Salmonella enterica*. Emerson et al. demonstrated that sEV released by *Salmonella*-infected macrophages, when administered to BALB/c mice, protected the animals from fatal infection [26]. Luo et al. demonstrated anti-tumor effects of exosomes derived from NK cells (NK-EXOs) in ovarian cancer [27]. Others have reported that NK-EXOs carry numerous proteins with cytotoxic properties, including perforins, granzymes, and granulysin, as well as cytokines such as IFN-γ or TNF-α, promoting the elimination of viral infections or limiting tumorigenesis [28,29]. sEV are involved in various other physiological processes such as vessel growth, tissue regeneration, or neuronal protection against oxidative stress [30].

In addition to the many physiological functions sEV modulate, they are also involved in mediating pathological mechanisms [31]; for example, their role in the development of various cancers, e.g., lung cancer [32,33], breast cancer [31], melanoma [34], hepatocellular carcinoma [35], and cervical cancer [36], is well documented. Additionally, sEV involvement in the promotion of viral infections has been described [37]. The relationship between sEV and oncoviruses in the context of tumor development has been of special interest. Oncoviruses, a group of viruses capable of causing cancer, are associated with 12% of all human cancers [38]. Currently, this group includes seven viruses with different taxonomic affiliations, namely, human T-cell lymphotropic virus-1 (HTLV-1), hepatitis C virus (HCV), human herpesvirus-8 (HHV-8), Epstein–Barr virus (EBV), Merkel cell polyomavirus (MCPyV), human papillomavirus (HPV), and hepatitis B virus (HBV) [38]. The mere fact of oncovirus infection alone does not determine the occurrence of cancer, reflecting the complex relationships between oncoviruses and tumors. Research indicates that these processes appear to be strongly linked to sEV.

## 2. Types of Interactions between sEV and Viruses

**Figure 1:**
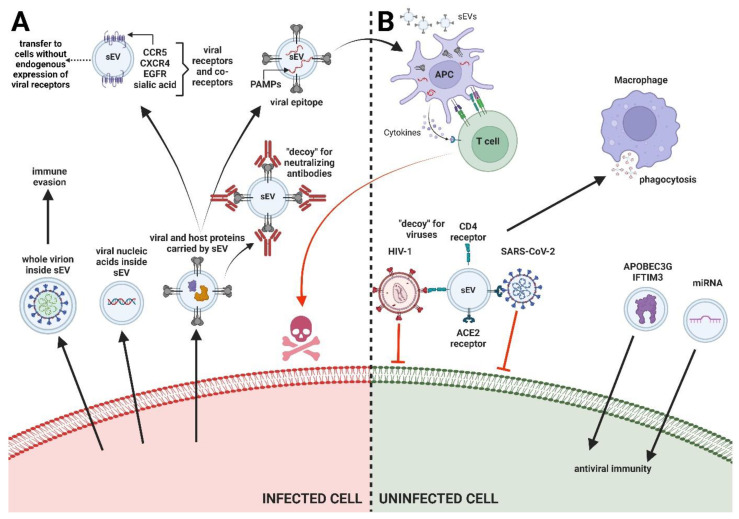
The influence of small extracellular vesicles (sEV) in viral infections. sEV can promote (**A**) or inhibit viral infections (**B**). The mechanisms underlying the sEV ability to facilitate viral infections are multifaceted, including the transmission of viral cargo, the delivery of entry receptors for the virus to new cells, and the binding of neutralizing antibodies. On the other hand, sEV antiviral effects are associated with the delivery of host restriction factors, such as APOBEC3G (apolipoprotein B mRNA editing enzyme catalytic subunit 3G) and IFTIM3 (interferon-induced transmembrane protein 3), as well as miRNAs that condition immunity to infection. sEV can also bind to virions (the „bait” mechanism), induce antiviral immune responses by delivering viral pathogen-associated molecular patterns (PAMPs) to antigen presenting cells (APCs), and promote the activation of natural killer cells (NK cells) and proliferation of CD4+ and CD8+ T cells. Created with BioRender.com.

Considering the remarkable ubiquity of sEV secretion by all types of cells, including immune cells, it should not be surprising that sEV influence the course of viral infections. sEV and viruses (especially enveloped viruses) exhibit many common features, both physical, chemical, and biological. Thus, the complete separation of sEV from virions during the isolation of the former is difficult or even impossible [39]. The mutual associations between sEV and viruses are also related to the interactions between the sEV biogenesis pathway and the viral replication cycle [40]. The effect of sEV on viruses can be twofold, acting as a “double-edged sword”. On the one hand, sEV can inhibit viral infection, e.g., by contributing to stimulation of antiviral immune mechanisms. On the other hand, they can promote viral infection e.g., by expanding the pool of cells susceptible to infection (transferring receptors for viruses or inducing receptor synthesis), increasing the efficiency of viral propagation and silencing the host immune response (see Figure 1) [41].

### 2.1. sEV and Inhibition of Viral Infections

#### 2.1.1. Transfer of Host Restrictive Factors

The antiviral effect of EVs (including sEV) can take various forms, depending largely on the specific virus. It often includes mechanisms that affect cells even before viral infection, preventing its occurrence [41]. An example is the transmission by sEV of the host restriction factors, i.e., proteins involved in the processes of innate antiviral immunity. In the case of human immunodeficiency virus 1 (HIV-1), sEV have been shown to transport APOBEC3G (apolipoprotein B mRNA editing enzyme catalytic subunit 3G) and thereby increase the resistance of target cells to HIV-1 infection in vitro [42]. On the other hand, recent studies indicate the potential involvement of APOBEC3 molecules in limiting the progression of tumors induced by human papillomaviruses (HPV), which indicates a negative role of sEV in the transmission of these molecules during infection [43,44]. However, there are still no conclusive studies confirming the role of APOBEC3-carrying sEV in oncogenesis. Kwasnik et al. [45] showed that infection with Influenza A virus (IAV) alters the profile of RNA in sEV secreted by infected cells by increasing the level of miRNA encoding RSAD2 (radical S-adenosyl methionine domain containing 2; virus inhibitory protein; viperin)—a host protein that inhibits viral replication of IAV [46], HIV [47], or HCV [48]).

#### 2.1.2. Binding and Neutralization of Virions by sEV Containing Entry Receptors

One of the ways for limiting viral infections is through increasing the presence of sEV that carry receptors recognized by viruses. By binding of virions to their surface, sEV can act as a “bait” and prevent the virus from entering cells [41]. This mechanism has been observed in different viruses, including the severe acute respiratory syndrome coronavirus 2 (SARS-CoV-2) and HIV-1 [49,50]. In the case of SARS-CoV-2, virions bind to sEV containing angiotensin-converting enzyme 2 (ACE-2). In HIV-1, the virus binds to CD4-coated sEV [51], although this effect might be negated by the viral protein Nef [52]. A similar mechanism has also been confirmed for the influenza virus. Suptawiwat et al. demonstrated that sEV secreted by bronchial epithelial cells carry sialic acid on the vesicle surface and can immobilize virions [53]. Previous research by Bedford et al. also suggested similar virion immobilizing effects of sEV isolated from mouse bronchial alveolar lavage fluid [54].

#### 2.1.3. miRNA Transfer for Enhancing Immunity to Infection

Recent research has emphasized the crucial role of microRNAs (miRNAs) in regulating immune responses against viruses [55]. These molecules are commonly found in the cargo of various EVs, including sEV. The antiviral activity of sEV containing certain miRNAs has been demonstrated for HCV where the inhibition of HCV infection by sEV derived from umbilical cord mesenchymal stem cells (uMSCs) was linked to the presence of Let-7f, miR-145, miR-199a, and miR-221 [56]. Similarly, sEV secreted by uMSCs have also been shown to play a role in reducing the replication of influenza A/B virus and human coronaviruses [57]. The specific role of miRNA-125b in suppressing influenza virus replication by inducing of interferon-stimulated genes (ISGs) has also been reported [57].

#### 2.1.4. Stimulation of Antiviral Immune Responses

sEV have a significant impact on both the innate and acquired immune responses to viruses. Several studies have demonstrated the ability of sEV to carry factors that modulate immune responses, particularly pro-inflammatory factors, including cytokines [58,59]. The study by Fitzgerald et al. reported that, of 33 plasma cytokines detected in soluble form, 11 were also secreted in the EV-bound form [60]. We have recently reported that in plasma of patients with HNSCC, 24/51 soluble cytokines were also carried in sEV, including IL6, TNFRII, IL-17a, IFNα, and IFNγ [61]. Additionally, Velandia-Romero et al. detected TNF-α, IL-6, complement protein C3, and metalloproteinase inhibitors in sEV released from dengue virus (DENV)-infected U937 macrophages and determined that DENV infection can increase the levels of certain inflammatory mediators in released sEV [62]. On the other hand, sEV can facilitate the recognition of viral pathogen-associated molecular patterns (PAMPs), such as viral proteins or nucleic acids, by transmitting them to appropriate pattern recognition receptors (PRRs), thereby promoting the initiation of antiviral responses [63,64]. The uptake of sEV containing viral antigens by antigen-presenting cells (APCs) can also contribute to a more efficient termination of infection by presenting viral antigens to effector cells [65,66].

### 2.2. sEV Effects on Viral Propagation

sEV play a critical role in the spread of viral infections by transporting bioactive viral particles, such as nucleic acids and proteins, or even whole virions. Furthermore, sEV can impair the host immune response or broaden viral tropism by delivering entry receptors to target cells and sensitizing them to infection. Here, we provide a comprehensive overview of the key mechanisms that contribute to the severity of viral infections mediated by sEV.

#### 2.2.1. Transport of Viral Cargo via sEV

##### En Bloc Transmission of Virions

For many years, the question of whether full virions are transported by sEV has been a topic of a debate and was considered controversial. The difficulties in separating virions and sEV, due to their similar physicochemical properties (such as size, density, structure, and content) have posed significant challenges and hindered the clear interpretation of test results [67]. When using the classical methods of sEV isolation, a possibility for co-isolation of virions should be considered. This requires the use of EM to confirm the presence of virions within sEV. Early research investigating the sEV potential for carrying full virions focused primarily on non-enveloped picornaviruses [68,69]. Feng et al. were the first to show that hepatitis A virus (HAV) can be released from cells inside sEV, taking on a form resembling an enveloped virus (*quasi*-enveloped HAV; eHAV), thereby confirming the existence of an alternative, non-lytic route of HAV exit from cells [68]. In the case of non-enveloped viruses, this mechanism may provide protection against the host’s immune response by concealing the virion within sEV [70]. The release of virions in sEV has been confirmed for viruses such as poliovirus [71], coxsackievirus B3 [72], hepatitis E Virus (HEV) [73], and rotavirus [74]. Given that enveloped viruses are already covered in a lipid bilayer, obtaining additional protection through sEV may seem unnecessary. However, it has been observed that even enveloped viruses use EVs (including sEV) to transport virions. Examples include the hepatitis B virus (HBV) [75] and HCV [76]. This strategy might be beneficial for the virus probably due to additional protection against the immune response, and it extends the range of potential target cells for the virus [77].

##### Transfer of Nucleic Acids

In viral infections, sEV can carry a wide array of different nucleic acids that are involved in the infection process. These nucleic acids can be categorized into three main types: full viral genome, mRNA, and non-coding RNAs [67]. The full viral genome, which consists of DNA or RNA (and comprises the entire genome of the virus), has been detected in EVs, including HIV-1 [78], HBV [79,80], and HCV [81]. Additionally, Mata-Rocha et al. detected part of the HPV-18 DNA genome in sEV secreted by HeLa cells with an integrated HPV-18 genome [82].

sEV can transport both mRNA that is a product of viral gene transcription and cellular mRNA encoding proteins that are important for the progression of viral infection [67]. This mechanism might also play a significant role in carcinogenesis caused by oncoviruses. If sEV carry mRNA transcripts that encode viral oncoproteins, the potential exists for the synthesis of these oncoproteins in target cells. Examples include mRNAs encoding the latent membrane protein (LMP-1) of the Epstein–Barr virus (EBV) [83], HBx of HBV [84], Tax of human T-lymphotropic virus (HTLV-1) [85], and E6/E7 of HPV [86]. A separate class of molecules that constitute the cargo of sEV are non-coding RNAs, among which miRNAs play a special role [67]. In many viral infections, sEV transport miRNAs encoded in the viral genome or specific host miRNAs that play a vital role in shaping the infection environment and whose expression level can be modified by the virus [87].

##### Transfer of Viral Proteins

The transmission of viral proteins is a fundamental mechanism by which sEV could influence the progression of an infection. This applies to both structural and non-structural proteins of the virus. In the case of structural proteins, numerous viral glycoproteins have been detected in sEV, such as Spike (SARS-CoV-2) [88], E1 and E2 (HCV) [89], Env (HIV) [90], and E (Zika) [91]. The presence of sEV coated with viral glycoproteins may challenge the humoral immune response, as they can capture neutralizing antibodies against viral glycoproteins and therefore prevent the targeting of true virions. This mechanism has been confirmed, for example, for the Zika virus (ZIKV) and SARS-CoV-2 [88,91].

In addition to structural proteins, sEV also transport non-structural and regulatory proteins of viruses, often with important functions. An example is the HIV Nef protein, which exhibits neurotoxic effects, and this effect is amplified by transport via sEV [92]. Of particular interest is also the transmission by sEV of viral oncoproteins such as LMP-1 (EBV) [93], HBx (HBV) [84], and Tax (HTLV-1) [94].

The role of sEV in infection may also be accomplished by the transport of certain host proteins. An example is the transmission of cellular receptors and coreceptors for the virus by sEV, which can lead to an increase in the pool of cells susceptible to infection. One of the first research papers to demonstrate the existence of such a mechanism is the publication by Mack et al., in which the authors showed the transfer of chemokine receptor type 5 (CCR5), i.e., a coreceptor for M-tropic strains of HIV, to cells without its endogenous expression [95].

### 2.3. Hijacking of sEV Biogenesis Pathways by Viruses

Viruses, as obligate parasites, rely on the host’s cellular machinery and metabolic pathways to complete their replication cycle [96]. The virus–host coevolution, which has lasted at least 3 billion years, has resulted in the development of mechanisms for controlling the host cell, ensuring the maximum propagation of new viruses and effective protection against the host’s immune system [97]. One such mechanism is the use of the EV biogenesis pathway, including sEV. Many viruses hijack the cellular machinery of EV biogenesis, such as the ESCRT and Rab-GTPase complex, which can be utilized to release virions and enclose them in a lipid envelope [37]. MVBs are employed by both enveloped viruses (e.g., HBV [98], DENV [99], SARS-CoV2 [100], HHV-6 [101,102], and HCMV [103]) and non-enveloped viruses (e.g., HAV [104], HEV [73], Enterovirus 71 [105], and Bluetongue virus [106,107]). In the case of some viruses, it is possible to utilize ESCRT to release virions from the cell without their entry into MVBs. An example of this is HIV-1, which acquires its lipid envelope in this process [108,109,110]. Other viruses, such as herpes simplex virus 1 (HSV-1), can use ESCRT to travel from the nucleus to the cytoplasm [111,112]. The use of sEV biogenesis pathways by viruses has been thoroughly summarized by Moulin et al. and is not further discussed here [37].

## 3. sEV Significance in Oncovirus Infections

Oncoviruses constitute a diverse group of viruses with differing taxonomic affiliations, structures, and replication cycles that have the potential to induce cancer. Although oncoviruses have the ability to induce cancer, this does not enhance their replication capacity, transmissibility, or viral load [113]. Carcinogenesis is not an integral part of the replication cycle of oncoviruses [114]. Thus, the development of cancer in response to induction by oncogenic virus appears to be more of a „biological accident” than an evolutionary adaptation [115]. In this context, sEV, as a key element of cell-to-cell communication, might play a crucial role in exacerbating the pathological effects of oncoviruses.

Efforts are ongoing to apply engineered sEV to new therapeutic strategies against oncoviruses. For example, a new form of therapy against HCV could include sEV loaded with anti-HCV miRNAs (let-7f, miR-145, miR-199a, and miR-221) [116]. In the case of HBV infection, sEV loaded with miR-574-5p may exhibit potential therapeutic effects [117]. In addition, the treatment of tumors induced by oncoviruses could be based on the use of sEV released by mesenchymal stromal cells (MSCs). The use of sEV derived from MSCs has many advantages over traditional cell therapies. By carrying cargo derived from MSCs, EVs provide paracrine effects similar to those mediated by MSCs, while also inducing low immunogenicity. Due to the smaller size of sEV compared to cells, there is also no risk of blood vessel blockage when administering sEV intravenously [118].

In this review, we summarize information on the role of sEV in the infections and pathogenesis of oncovirus-induced cancers. The discussed mechanisms of sEV-induced alterations in oncoviral infections are summarized in Table 1.

### 3.1. RNA Oncoviruses

#### 3.1.1. Retroviruses

##### HTLV

In light of recent research, the significant role of sEV in HTLV-1 infection is becoming increasingly evident. Although intact HTLV-1 virions have not yet been detected in sEV, these vesicles are well-documented for carrying viral proteins (Tax and HBZ) and transcripts (*tax*, *hbz*, and *env* mRNA) [85,119], and recent reports also indicate the detection of genomic RNA [120]. HTLV-1 infection also alters the host protein expression profile carried by sEV [119]. Presumably, mechanisms related to the viral replication cycle promote the selective packaging of certain host proteins into sEV, as indicated by comparative proteomic analysis conducted by Jaworski et al. [119]. On the other hand, sEV released from infected cells promote cell-to-cell contact by carrying adhesion factors (ICAM-1 and LFA-1), which is crucial for HTLV-1 propagation [121]. The virus spreads 10,000 times more effectively this way compared to the release of free virions [122].

The carcinogenic effect of HTLV-1 infection appears to be amplified by sEV interactions. sEV inhibit apoptosis in target cells and transfer miRNA that promote oncogenic transformation in leukemias (miR-21 and miR-155) [123]. sEV from ATLL (adult T cell leukemia/lymphoma) cells not only carry the oncogenic Tax protein but also VEGF (vascular endothelial growth factor), a proangiogenic factor [123]. They induce morphological changes in MSCs, promote their proliferation, and activate the expression of genes associated with angiogenesis [123].

#### 3.1.2. Flaviviruses

##### HCV

The relationship between sEV, HCV infection, and carcinogenesis is still not fully comprehended; however, recent research indicates that sEV have a significant impact on the effectiveness of HCV infection and are crucial in the transmission process between hepatocytes [81]. Released from infected cells, sEV carrying HCV virions can cause effective infections, similar to free virions, and are better protected against environmental influences [81,124]. An additional aim of the virus is to evade detection by the immune system by encapsulation within sEV, thus increasing the likelihood of infecting target cells [125]. Giannessi et al. proposed an interesting theoretical concept suggesting that sEV may facilitate the spread of infection by non-infectious, defective virions, for example, by allowing entry into cells by surface glycoprotein-defective virions that utilize the presence of cellular receptors on the surface of sEV [126,127].

Naïve liver cells can be infected by HCV RNA-sEV, secreted from HCV-infected cells, in a receptor-independent pathway, even in the presence of blocking antibodies directed against HCV receptors [125]. 

**Table 1:** The key mechanisms of sEV-mediated alterations that impact oncovirus infections.

Virus	Active Molecule in sEV		Mechanism	Effect	Refs.
	Viral Cargo	Cellular Cargo			
**RNA oncoviruses**					
HTLV-1	Tax, mRNAs (tax, hbz, env), genomic RNA	Proinflammatory cytokines, miR-21, miR-155,VEGF, CD45,CD43, ICAM-1, LFA-1	Provide protection for virions against neutralization and environmental factors	Promote infection	[119]
			Prevent Fas-mediated apoptosis by inducing the cFLIP and NFkB signaling pathways	Increase survival of target cells, inhibit apoptosis	[119]
			Transfer adhesion factors to the target cell	Increase cell-to-cell contact and agglutination potential	[121,128]
			Carry angiogenic factors and promote the proliferation of target cells	Promote tumor progression	[123]
HCV	intact virions, E2 protein, genomic RNA, viral RNAs	miR-122, miR-21, miR-34a	Transfer of intact virions; enable the infection of target cells by defective virions (e.g., without surface glycoproteins)	Increase infection efficiency	[126]
			Provide physical protection against the neutralization of virions	Impart resistance to neutralization by anti-HCV neutralizing antibodies	[81]
			Modify gene expression in target cells by transferring different miRNAs	Create favorable tumor environment	[129]
			Transport miR-122 to target cells, as well as HCV-RNA in a complex with miR-122-Ago2-HSP90	Promotes HCV replication	[125]
			Transport miR-21 to target cells	High levels of miR-21 expression in plasma sEV are correlated with clinical-pathological features of HCC	[130]
			Transport of miR-34a	Induction of apoptosis	[131]
**DNA oncoviruses**					
HBV	intact virions, viral DNA, viral RNA, proteins (HBsAg, HBcAg, HBeAg, HBx, P pro-tein), HBV-miR-3	miR-122, miR-21, miR-29, miR-135a-5p	Transfer of intact virions	Facilitate virus propagation	[75]
			Transport of miR-135a-5p that alters gene expression in the target cell	Exhibit antiapoptotic activity towards cancer cells	[132]
			Decrease of RIG-I expression in NK cells and reduction of NFkB pathway activation	Inhibition of proliferation, cytotoxic activity and interferon ɣ production of NK cells	[133]
			Transport of HBV-miR-3	Promotion of angiogenesis	[134]
			Transport viral HBx protein that lowers the level of expression of antitumorigenic miR-122	Promote tumorigenesis	[135]
			HBV infection increases the expression of miR-21 and miR-29 in sEV, which alters gene expression in target cells	Inhibition of IL-12 release by macrophages and dendritic cells	[132]
HPV	viral DNA, viral proteins (E6, E7), E6/E7 mRNA	apoptosis inhibitors (surviving, XIAP, c-IAP1, c-IAP-2, ML-IAP), immunoregulatory molecules (CD276, CD47), calmodulin, MUC16, SIRPA	Transfer of viral oncoproteins E6/E7	Promotion of tumorigenesis	[86]
			Transport of MUC16 and SIRPA proteins	Induce epithelial-mesenchymal transition	[36]
			Generate endoplasmic reticulum stress in target cells and decrease expression of proteins associated with tight junctions (ZO-1, claudin-5)	Promotion of metastasis and tumor progression	[36]
			Transport of immunomodulatory factors	Stimulate dendritic cell (DC) maturation and sustain their function	[136]
			Transport of apoptosis inhibitors (surviving, XIAP, c-IAP-1, c-IAP-2, ML-IAP)	Inhibition of apoptosis of cancer cells	[137]
EBV	LMP-1, LMP-2A, gp350, viral RNA, viral miRNA, EBERs	HIF1α, Galectin-9, EGFR	Transfer oncogenic viral proteins LMP-1 and LMP-2A	Induction of tumorigenesis	[93,138]
			Transport of HIF1α factor	Induction of angiogenesis	[139,140]
			Increasing level of EGFR expression	Promotion of metastasis	[141]
			Viral protein LMP-1 modify content of cargo of sEV to increase expression proteins involved in EBV infection	Increase infection efficiency	[142]
			miRNAs modulate viral and host gene expression	Facilitate infection and immune evasion	[142,143]
KSHV	viral RNA, viral miRNA	IL-1β, IFI16	Transported viral miRNAs affect cell metabolism and tumorigenesis	Creation of microenvironment favorable to tumor development	[144,145]
			Removal of immune-inducing factors from the cell)	Facilitate immune response evasion	[146,147]
			Complement system activation	Promotion of long-term latency by activating NFκB pathway	[148]
MCPyV	viral oncoproteins, ALTO	miR-375	Transport of viral oncoproteins and circular RNA (ALTO)	Increase expression of host genes associated with pathogenesis of MCPyV infection	[149,150]

VEGF: vascular endothelial growth factor; LFA-1: lymphocyte function-associated antigen 1; XIAP: X-linked inhibitor of apoptosis; c-IAP1-2: cellular inhibitor of apoptosis protein 1-2; ML-IAP: melanoma inhibitor of apoptosis; LMP-1: Epstein–Barr virus latent membrane protein 1; EBERs: Epstein–Barr virus-encoded small RNAs; EGFR: endothelial epidermal growth factor receptor; ALTO: alternative large T antigen open reading frame protein.

Bukong et al. detected HCV RNA in sEV that was found to be associated with Ago2 (Argonaute 2), HSP90 (heat shock protein 90), and miR-122, which may have a stabilizing function with regard to HCV RNA, as suggested by other studies [125]. This finding may explain the ineffectiveness of immunological therapies with anti-HCV-receptor antibodies and indicate the potential of alternative therapies such as miR-122 inhibitors or HSP90 inhibitors [151].

The pathogenesis of HCV-associated cancers is primarily mediated by the production of sEV, which creates a favorable environment for infection and tumor development [129]. Similar to other cancers, miRNAs are a particularly important class of bioactive molecules transported by sEV for the initiation and progression of hepatocellular cancer (HCC). An important example is miR-122, which is highly expressed in the liver and can be found in sEV [125,152]. This molecule participates in promoting HCV replication. Wang et al. discovered that high levels of miR-21 expression in plasma sEV are correlated with clinical-pathological features of HCC such as cirrhosis and tumor staging [130]. Badami et al. have reported an upregulation of miR34a expression in the HCC cell line Huh7.5 after HCV infection. The same study found that sEV secreted by infected cells also demonstrated increased miR34a expression, and when these sEV were applied to Huh7.5 cells, they induced apoptosis [131]. Other studies have demonstrated a correlation between the level of miR34a overexpression and the development of HCV-induced liver cirrhosis [153].

### 3.2. DNA Oncoviruses

#### 3.2.1. Hepadnaviruses

##### HBV

**Figure 2:**
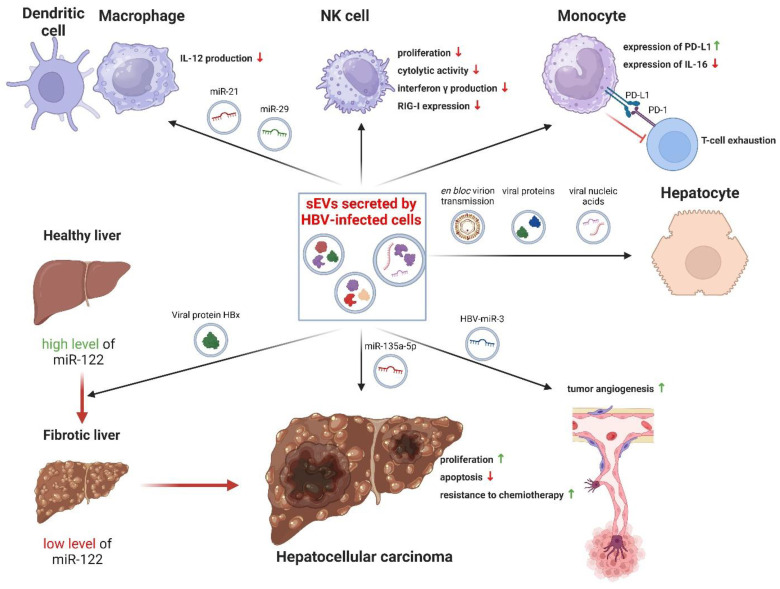
Role of sEV in HBV infection and the development of HBV-induced liver disease. sEV secreted from HBV-infected cells transport bioactive cargo and may promote progression of HBV-dependent HCC. Created with BioRender.com.

Through various mechanisms, sEV promote HBV infection and the onset of HBV-related diseases (including HCC). The transmission of viral cargo by sEV secreted from HBV-infected cells is the main mechanism that promotes HBV propagation (see Figure 2) [133,154]. Recently, Wu et al. demonstrated the presence of full HBV virions in sEV for the first time [75]. There are several reports of the transfer of viral protein (HBx, HBsAg, HBeAg, HbcAg, and P protein), DNA (rcDNA and cccDNA), and RNA (XBx RNA, HBs/p RNA, and HBV-miR-3) in sEV released by infected cells [84,133,155]. In addition, studies using mass spectrometry and miRNA sequencing techniques have revealed differences in the expression profiles of proteins and host miRNAs in sEV derived from normal hepatocytes (THLE2 and THLE3) and HBV-infected hepatocytes (SNU-423 and SNU-182) [156]. According to functional analyses, many of these proteins and miRNAs are involved in the viral replication cycle, sEV biogenesis, and carcinogenesis, which may indicate an important role of sEV in the development of HCC induced by HBV infection [156].

Released from HBV-infected cells, sEV undergo endocytosis by monocytes, resulting in elevated PD-L1 levels in these cells. This observation is consistent with the high levels of PD-L1 expression in the monocytes of patients with chronic hepatitis B [157,158]. PD-L1 can inhibit the immune response against cancer by binding to PD-1 on T cells and promoting the exhaustion of these cells [159]. Additionally, sEV containing viral cargo can negatively affect other immune cells, such as NK cells [133]. In the study of Yang et al., the interaction of sEV containing HBV cargo with NK cells resulted in the disruption of functions such as proliferation, cytotoxic activity, and interferon ɣ production, and also caused a decrease in the expression of RIG-I receptors and inhibition of NF-κB and MAPK signaling pathways [133].

It has been shown that HBV proteins also affect the level of cellular miRNA expression in sEV, causing an increase in the expression of some miRNAs and a decrease in the expression of others, and thus can actively modulate the microenvironment, promoting the progression of CHB to HCC [154]. One example is the role of the viral HBx protein, which exhibits an oncogenic effect by lowering the expression level of miR-122, that is, miRNA involved in inhibiting the development of HCC by binding to genes responsible for proliferation [135]. HBx may also interact with the biogenesis pathways of sEV, leading to a change in their protein profile [84,160]. Moreover, the viral HBcAg protein increases the level of miR-135a-5p in sEV, which has a protective effect against apoptosis and also increases the proliferation of HCC cells and their resistance to chemotherapy [161]. Kouwaki et al. observed that HBV infection resulted in an increased expression of miR-21 and miR-29 in sEV, leading to the inhibition of IL-12 release by macrophages and dendritic cells [132].

#### 3.2.2. Papillomaviruses

##### HPV

One of the first experiments confirming the transmission of cargo by sEV that could promote HPV-dependent carcinogenesis concerned the presence of survivin—a protein with anti-apoptotic activity [162,163]. Moreover, the presence of other apoptosis inhibitors, such as XIAP, c-IAP1, c-IAP2, and ML-IAP, has also been confirmed in sEV released from HPV-infected cells [164]. Honegger et al. found that sEV secreted by HeLa HPV-infected cells contain different miRNAs, and their composition depends on the endogenous expression of E6/E7 in infected cells [165]. It was observed that sEV secreted by oropharyngeal cancer (OPC) cells affected non-cancerous mammalian HPV (−) epithelial cells by inducing a epithelial-mesenchymal transition (EMT) and making them more invasive. HPV-16 E7 was also detected in previously uninfected HPV cells [166]. The main mechanism of oncogenesis conditioned by viral proteins E6/E7 was partially understood several decades ago and is based on the blocking of the most important cellular tumor suppressors by these proteins. The E6 protein blocks p53 by stimulating its degradation, while E7 binds to pRB, causing its inactivation and proteolysis, and releasing the E2F factor at the same time [167,168].

Ludwig et al. conducted a comparative proteomic analysis of sEV from HPV-infected and not-infected head and neck cancer (HNC) patient-derived cells. The results revealed that E6/E7, p16, and survivin proteins were detected only in HPV (+) cell-derived sEV, while multiple immunomodulatory proteins (TGF-β, FasL, OX40, OX40L, and HSP70) were detected in sEV from both HPV (+) and HPV (−) cells [169]. Interestingly, further studies revealed that HPV (+) cell sEV caused dendritic cell (DC) maturation and sustained their function, unlike HPV (−) cell sEV exhibiting the opposite effect [169]. This may explain previous observations indicating that HPV-infected HNC patients have a better prognosis than non-infected patients and respond better to some forms of therapy [136].

#### 3.2.3. Herpesviruses

##### EBV

The presence of various viral elements in sEV, i.e., proteins, viral mRNA, miRNA, or Epstein–Barr virus-encoded small RNAs (EBERs) has been documented [170,171]. Among the EBV proteins found in sEV, LMP-1 and LMP-2A appear to be particularly important [93]. Latent membrane protein 1 (LMP-1) is a major EBV oncoprotein that is involved in B cell transformation by binding to TRAF6 and inducing signaling pathways typically triggered by CD40L binding to the CD40 receptor [172]. This leads to enhanced B cell viability, cell growth, and the inhibition of apoptosis [172]. Proteomic studies by Nkosi et al. showed that LMP-1 has the ability to modify the cargo of sEV secreted from infected B lymphocytes, increasing the content of proteins involved in EBV infection, endocytosis, apoptosis, and the MAPK and NF-Kb signaling pathways, as well as adhesion molecules [93]. Under the influence of LMP-1, the level of proteins involved in the cellular organization of organelles and catabolic processes in sEV was reduced [93]. In addition to modifying the protein profile of secreted sEV, LMP-1 may also enhance the process of sEV secretion [139]. Research in recent years has increasingly highlighted that LMP-1 is involved in the development and progression of various types of cancer, including gastric cancer [173], nasopharyngeal carcinoma (NPC) [138], and lymphoma [174]. The importance of LMP-1-carrying sEV in promoting different cancers is becoming evident [138].

In addition to viral factors, sEV released from EBV-infected cancer cells can carry cellular factors that promote tumor progression. This effect has been confirmed, for instance, in NPCs, where sEV from EBV (+) cells transport the promethastatic factor HIF1α [139]. Moreover, sEV from EBV (+) NPC cells may promote immune evasion, thereby ensuring uninterrupted tumor growth [142]. Galectin-9, which interacts with Tim3 and induces apoptosis in T cells, has also been identified in sEV secreted by EBV-infected NPCs [143]. NPC is characterized by a high level of EGFR expression, which in turn can be induced by LMP-1, and LMP-1 simultaneously promotes the release of EGFR into sEV [141].

##### KSHV

In the course of its replication cycle, KSHV exploits various metabolic pathways of the host cell, ultimately resulting in the formation of a microenvironment favorable for virus propagation and the evasion of the host’s immune response [146]. The manipulation of sEV biosynthesis pathways is one such mechanism that the virus utilizes [146]. In fact, KSHV-infected cells secrete a significantly higher number of sEV than uninfected cells [148]. Research has shown that sEV secreted by KSHV-infected cells contain IL-1β and IFI16 (interferon gamma inducible protein 16), two important factors involved in immune mechanisms [147]. This may indicate that KSHV uses sEV to remove immune-activating factors from the cell [146,147]. Surprisingly, according to results from Jeon et al., sEV from KSHV-infected epithelial cells can activate the complement system in both infected cells and neighboring cells [175]. Furthermore, this sEV-dependent activation of the complement system not only fails to block infection, but promotes persistent KSHV latency and increases the survival of infected cells, particularly by activating the NFkB pathway [175].

As in the case of EBV, KSHV also encodes viral miRNAs in its genome, which can affect host gene expression, immune processes or tumorigenesis [176]. It is not surprising that these viral miRNAs are also present in sEV secreted from KSHV-infected cells. Chugh et al. confirmed the presence of all KSHV miRNAs in sEV isolated from plasma of infected patients [144]. KSHV miRNAs can affect cell metabolism by adjusting it to maintain long-term KSHV latency. By decreasing mitochondrial biogenesis and inducing glycolytic pathways, KSHV miRNAs result in reduced oxygen consumption, increased glucose uptake, and increased lactate secretion, reminiscent of the KSHV-induced Warburg effect [177,178]. Other functions of individual KSHV miRNAs in the context of KSHV replication cycle have been described in detail in Dass et., therefore it will not be discussed here in more detail [179].

#### 3.2.4. Polyomaviruses

##### MCPyV

The Merkel cell polyomavirus (MCPyV) is the latest discovered oncovirus, which was described by Feng et al. in 2008 [180]. Due to its relatively late discovery, this virus is the least understood oncogenic virus and there is limited research on the role of sEV in MCPyV infection. Konstantinell’s secretome studies revealed that sEV secreted by MCPyV-infected MCC cancer cells carry viral oncoproteins. An analysis of the sEV proteome secreted by MCPyV-infected and non-infected MCCs showed significant differences [181]. Yang et al. detected circular RNA encoding the viral protein ALTO (alternative large T antigen open reading frame) in sEV secreted by MCPyV-infected MCC cell lines. It has also been demonstrated that ALTO can promote the expression of host genes associated with the pathogenesis of MCPyV infection [149].

Research has shown that sEV released by MCCs also contain miRNAs. Fan et al. detected the presence of high levels of miR-375 [182]. Fan et al. demonstrated that the horizontal transfer of miR-375 to fibroblasts via sEV resulted in the polarization of these cells to cancer-associated fibroblasts (CAFs). This indicates the role of miR-375 in MCC to create a pro-cancer microenvironment [182].

## 4. Conclusions

Research conducted in recent years has recognized sEV as key factors in the pathogenesis of cancer induced by oncoviruses. sEV carry a wide spectrum of bioactive macromolecules involved in both the development of viral infections and in carcinogenesis. However, there still exists a lack of detailed information about the mechanisms that govern the interactions between viruses, sEV, and cancer. Research in this area is especially important for the design of future anticancer therapies, which should take into account the pathologic mechanisms mediated by sEV. Future anti-viral therapies might also be able to take advantage of targeting sEV, which promote viral infections

## Data Availability

Not applicable.
